# Preventable trauma deaths: from panel review to population based-studies

**DOI:** 10.1186/1749-7922-1-12

**Published:** 2006-04-11

**Authors:** Osvaldo Chiara, Stefania Cimbanassi, Alessio Pitidis, Sergio Vesconi

**Affiliations:** 1 Niguarda Ca'Granda Hospital, Trauma Team, Post-Graduate School of General Surgery, University of Milano, Italy; 2 National Institute of Health of the Ministry of Health, Rome, Italy; 3 Niguarda Ca'Granda Hospital Division of Anesthesiology and Intensive Care, Post-Graduate School of Anesthesiology and Intensive Care, University of Milano, Italy

## Abstract

Preventable trauma deaths are defined as deaths which could be avoided if optimal care has been delivered. Studies on preventable trauma deaths have been accomplished initially with panel reviews of pre-hospital and hospital charts. However, several investigators questioned the reliability and validity of this method because of low reproducibility of implicit judgments when they are made by different experts. Nevertheless, number of studies were published all around the world and ultimately gained some credibility, particularly in regions where comparisons were made before and after trauma system implementation with a resultant fall in mortality. During the last decade of century the method of comparing observed survival with probability of survival calculated from large trauma registries has obtained popularity. Preventable trauma deaths were identified as deaths occurred notwithstanding a high calculated probability of survival. In recent years, preventable trauma deaths studies have been replaced by population-based studies, which use databases representative of overall population, therefore with high epidemiologic value. These databases contain readily available information which carry out the advantage of objectivity and large numbers. Nowadays, population-based researches provide the strongest evidence regarding the effectiveness of trauma systems and trauma centers on patient outcomes.

## Introduction

Increasing expectancy of health care provided by public or private institutions have promoted a number of programs under the generic title of quality assessment. In recent years these programs have been increasingly directed toward optimization of care based on accepted standards with an attention to costs containment. Emergency trauma care has not been excluded from this process of evaluation. Initially, during the 1960s and 1970s it became apparent, for many reasons, that trauma care in the North America was suboptimal and even inadequate. On the basis of this collective feeling, many surgeons decided that they should evaluate quality of care and translate it into health policy changes.

In the classic description trauma deaths have a tri-modal distribution [1,2]. *Immediate deaths *occur immediately after trauma and are due to non-salvageable injuries like rupture of the heart or great vessels. *Early deaths *occur during the first six hours and are due to evolving conditions like hemorrhagic injuries of abdominal organs or expanding intracranial mass lesions. *Late deaths *occur after days or weeks and are due to sepsis and multiple organ failure. Early deaths are commonly considered preventable because they may be avoided if injuries are promptly identified and treated. Therefore, surgeons turned to the traditional mortality and morbidity methodology, frequently used in modern teaching to critically analyze failures of care, with particular attention to deaths of the second peak. Reviews of hospital charts by panel of experts, comparison of observed survival with probability of survival obtained from national registries, and population-based epidemiological studies are the three models which have been used during the past twenty years for quality assessment of trauma care. Following paragraphs summarize methodological aspects and results of various preventable trauma deaths studies.

## Panel studies of preventable trauma deaths

The preventable death rate (PDR) is the proportion of deaths judged to be preventable if optimal trauma care has been delivered. A trauma death defined as preventable needs to meet three criteria: *1*. the injuries produced by trauma and its sequelae must be survivable, *2*. the care which has been delivered must be suboptimal when compared with standards, *3*. the identified errors in the delivery of care have directly or indirectly contributed to patient outcome [3]. The methodology used to establish PDR of a given population has been at the beginning the review of charts (usually autopsy studies) by one or two independent personalities. These early studies were criticized because of subjectivity based in many instances on the judgment of a single reviewer of autopsy reports, while hospital administrators and doctors were reluctant to have their charts analyzed. Subsequently, outcomes of trauma care and performance of emergency organizations were reviewed by multidisciplinary panels of experts with a larger effort to introduce more explicit criteria of judgment. The most used method has been to examine the relationship between deaths and appropriateness of delivered care, when compared to given standards. Table 1 shows the most used standards to evaluate adequacy of trauma care. Traditionally, evaluation of preventability of a trauma death has been translated into three categories[4]:

**Table 1:** Standards of adequacy in trauma care. Pre-hospital time is considered from dispatch to hospital arrival; LOC: loss of consciousness; DPL: diagnostic peritoneal lavage; US: ultrasound; ET: emergency department thoracotomy; RH: retroperitoneal hematoma

**Time errors**	**Mismanagement**	**Missed injury**
1. pre-hospital time > 30 minutes 2. admission – OR time > 2 hours in patient requiring laparotomy or thoracotomy 3. admission-OR time > 4 hours in patient requiring craniotomy for epidural or subdural hematoma 4. transfer to a higher level hospital > 6 hours after initial hospital arrival	1. management not appropriate to ATLS guidelines during resuscitation (pre & in-hospital) and therapeutic or diagnostic decision contrary to available data 2. lack of airway control in LOC patient 3. lack of intravenous fluids in exsanguinating patient 4. lack of external bleeding control 5. lack of immobilization 6. lack of pleural decompression when requested 7. lack of thoracostomy tube in hemo-pneumothorax 8. lack of DPL or US or laparotomy in hemoperitoneum with unstable hemodynamics 9. median sternotomy in patient requiring ET 10. laparotomy in RH from closed pelvic fracture without associated abdominal injury 11. lack of CT-scan in GCS < 13 within 2 hours	an injury important in patient demise missed because of misinterpretation or inadequacy of physical examination or diagnostic procedures.

a. the *non-preventable death *(NP), when injuries are not survivable and not currently curable or reversible. Any patient dead with at least one injury scored 6 at the abbreviated injury scale (AIS) [5], like a rupture of the heart or a crash injury of the head, belongs to this category.

b. the *possible preventable death *(PP), when injuries are severe, but currently curable or reversible under optimal circumstances. For example, a subdural hematoma is treatable if evacuated in a short time in a hospital with a neurosurgical service. If the patient is initially admitted to a facility without neurosurgery and dies while waiting for the transfer, this death may be considered PP.

c. the *definitely preventable death *(DP), when injuries are curable or reversible under the existing facilities. Patient demise is due to injuries easily treatable in the place where patient has been admitted, as a death for an unrecognized ruptured spleen inside a hospital furnished of a general surgical service.

Critical to the reliability of these studies is the case mix of the study population, the information available for review, the composition of panel and process used to make final judgment.

Ideally, all trauma deaths occurred in a definite geographical region should be considered because magnitude of PDR may be influenced by a selected case population.

Bias may also derive from the amount of available data: pre-hospital reports, hospital records and autopsy study should be all considered for consultation (compared to clinical records autopsy reports often describe more severe injuries thus decreasing preventability).

Composition of the panel may influence results of the discussion: trauma or general surgeon, orthopaedic surgeon, neurosurgeon, anaesthesiologist, emergency physician, epidemiologist, pathologist, nurses, have been usually included in panels to provide the range of clinical expertise necessary to evaluate all the aspects of patient care.

Regarding the process of evaluation, four types of judgment methods have been followed in various studies[6]:

*1*. independent review of cases by each panelist and the preventability assigned only on the majority opinion of experts

*2*. independent review followed by a panel consensus for deaths considered preventable at least by one panelist

*3*. modified independent review followed by a panel consensus in cases where there is not a majority opinion

*4*. independent review and the preventability assigned only to cases with unanimous decision by panelists.

Methods 1 and 4 are more conservative and provide an estimate of the lower bound of PDR. Methods 2 and 3 allow a panel discussion where panelists influence each other increasing the number of deaths judged to be preventable and providing the upper bound of PDR. Analysis of reliability of panel studies includes evaluation of agreement between single panelists and between different panels. Kappa-type statistics [7] have been used to assess the amount of agreement with k=1 when there is complete agreement and k=0 when agreement is only by chance. Agreement in published studies was extremely variable, being higher for non central nervous system deaths and for DP deaths, but generally not exceeding the 50%. In general, physicians agreed about the nature of inadequacy of care (i.e. delay in diagnosis, delay in surgery), but disagreement concerned the extent to which inadequacy of care contributed to death.

Owing to these limitations and because of subjectivity involved in the methodology, panel studies represent structured case series studies and are classified as Class III evidence [8]. Table 2 shows the most important published studies about preventable trauma deaths. These investigations, when properly designed using predefined standards of care which are compared with delivered treatment, may generate information regarding the compliance of the system under study with the generally accepted principles of appropriateness of trauma care [9]. In general, panel studies evaluated a number of deaths from 42 to 255, showing a PDR from 35% to 45% in absence of organized trauma care with a reduction to 15%–20% after trauma system and trauma center implementation [9-15]. Two italian studies in different urban areas without an organized trauma care showed respectively a PDR of 37% and 43%[9,15]. Errors and delays during the first phases of in-hospital assessment and care were identified in these researches as the main failures of treatment.

**Table 2:** Panel studies of preventable trauma deaths

**Author**	**Journal and year**
Van Wagoner FH	J Trauma, 1: 401, 1961
Moylan JA, et al.	J Trauma, 16: 517, 1976
FoleyRW, et al.	J Trauma, 17: 611, 1977
Detmer DE, et al.	J Trauma, 17: 592, 1977
West JG, et al.	Arch Surg, 114: 455, 1979
Baker CC, et al.	Am J Surg, 140:144, 1980
Neumann TS, et al.	Am J Surg, 144: 722, 1982
Lowe DK, et al.	Am Surg, 23: 503, 1983
West JG, et al.	Arch Surg, 118: 740, 1983
Reines HD, et al.	Am Surg, 49:203, 1983
McCoy, et al.	J Pediatr Surg, 18: 505, 1983
Certo TF, et al.	J Trauma, 23:559, 1983
Krob MJ, et al.	Ann Emerg Med, 13:891, 1984
Ottosson A, et al.	JAMA, 251:2668, 1984
Ramenofsky ML, et al	J Trauma, 24:818, 1984
Spain DM, et al.	Am J Publ Health, 74:1122, 1984
Cales RH	Ann Emerg Med, 13: 1, 1984
Baker CC, et al.	Am J Surg, 149:453, 1985
Cales RH, et al.	Jama, 254: 1059, 1985
Shackford SR, et al.	J Trauma, 26: 812, 1986
Kreis D, et al.:	J Trauma, 26: 649, 1986
Shackford SR, et al.	J Trauma, 27: 866, 1987
Rivara FP, et al.	JAMA, 261:566, 1989
Guss DA, et al.	Ann Emerg Med, 18: 1141, 1989
Campbell S, et al.	Am Surg, 55: 478, 1989
Webb GL, et al.	Am J Surg, 159: 377, 1990
Cayten CG, et al.	Ann Surg, 214: 510, 1991
Thoburn E, et al.	J Emerg, Med 11: 135, 1993
Stocchetti N, et al.	J Trauma, 36: 401, 1994
Esposito TJ, et al.	J Trauma, 39: 955, 1995
Maio RF, et al.	J Trauma, 41: 83, 1996
Chiara O, et al.	Injury, 33: 553, 2002
Esposito TJ et al.	J Trauma, 54: 663, 2003

## The TRISS method

**Figure 1:**
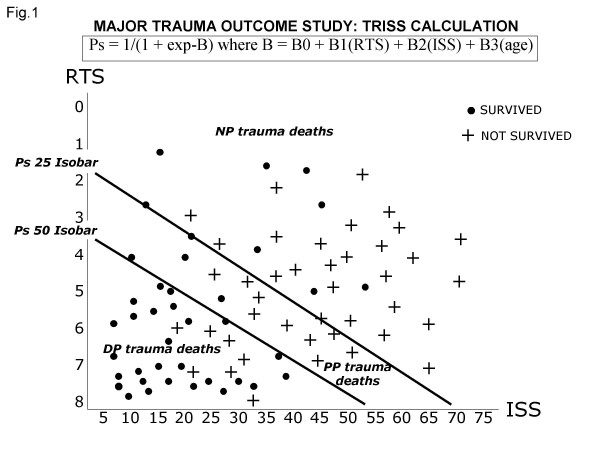
A graphic representation of TRISS calculation of probability of survival (Ps). B0-B3 are coefficients derived from the major trauma outcome study. Isobars of Ps=0.25 and of Ps=0.50 are shown. DD: definitely preventable; PP: possible preventable; NP: not preventable

Other studies compare observed survival with expected probability of survival derived from large specialized registries. The popular TRISS method calculates probability of survival (Ps) of a trauma patient from anatomy of injury scored with Injury Severity Score (ISS), physiologic severity at admission described by Revised Trauma Score (RTS), age and mechanism of trauma, as described in fig. 1. Coefficients B0-B3 are derived from Major Trauma Outcome Study, a registry of several thousands of trauma patients used to generate predicted adult death rates[16,17]. The use of TRISS formula removes subjectivity in the calculation of Ps and it has been suggested [13] to consider DP the deaths occurred with a Ps higher than 0.50 and PP the deaths occurred with a Ps between 0.50 and 0.25. Deaths in patients with a calculated Ps less than 0.25 have to be considered NP.

It is possible to obtain a graphic representation of DP, PP and NP deaths (fig. 1): patients who die with not severe injuries (low ISS and high RTS), are easily identified in the lower-left part of the panel and these unexpected deaths may be analyzed by experts in a quality assessment program [18-22]. In general, these studies show an increased observed survival, compared with TRISS calculated Ps, after the organization of trauma systems and trauma centers. A limitation of this method may be inadequate coding of ISS and RTS and underestimation of concomitant chronic diseases. Moreover, the collection of data for MTOS (which ages more than 15 years) is not population-based and the norms derived reflect the standards of trauma centers that voluntary participated to that study and not international norms. Because of these considerations also registry comparison studies are classified as Class III evidence [23].

## Population-based studies

Earliest trauma systems analyses were limited to seriously injured patients treated in trauma centers who constitute only 15–20% of the population of injured patients treated in the hospitals of a nation. From the end of last century there was a call for trauma systems to be inclusive. Inclusive trauma systems intend to benefit all injured patients requiring hospital treatment and the benefit should start in the pre-hospital care, should continue during hospital care and should conclude with the phase of recovery and rehabilitation [24]. The wide scope of an expected benefit to all the population in terms of decreased preventable deaths suggests the need of studies based on representative samples of all patients in a region. Population-based studies include data derived from all injured patients of a country, those who die before hospital arrival, those hospitalized in trauma centers and non-trauma centers and those treated in rehabilitation centers. These studies use representative databases such as death certificates, police reports data, state registry of fatalities after motor vehicle collisions, hospital discharge data with injuries identified by International Classification of Diseases, Ninth Edition, Clinical Modification (ICD-9-CM). These databases differ from trauma registries in several ways. Trauma registries contain greater detailed information but this is offset by the limitation of including only most serious patients treated at trauma centers and only after the implementation of trauma systems. On the contrary, population based data have been recorded for years, contain readily available, alphanumerical-coded information and allow low-cost analyses[25].

For example, in table 3 the incidence rate of trauma deaths in Italy during 2002 calculated from death certificates (which are required by law for all decedents) is summarized. All national deaths due to intended or unintended injury are included and these numbers may be easily used for epidemiologic studies comparing regions with and without implemented trauma systems or periods before and after system implementation. Trauma death in Italy has an incidence rate 35.3 × 100,000 inhabitants/year (total: 20,332). Hospital trauma deaths from discharge registry (table 4) are 17.1 × 100,000 (total: 9,871): therefore, more than 50% of trauma deaths in Italy occur before hospital arrival. Trauma death incidence rate in the San Diego County, which has a mature trauma system, is 24.0 × 100,000 inhabitants/year and pre-hospital deaths are 40.5%[26]. It is possible to emphasize that more than 10 × 100,000 inhabitants of Italian trauma deaths could be prevented especially during pre-hospital care with the implementation of an organized trauma system.

**Table 3:** Trauma death in Italy, year 2002: incidence rate × 100,000 inhabitants/year. Deaths are categorized for age, sex and main diagnosis. Vert. Column: spine injuries with or without cord injury. Multiple NOS: multiple internal injuries not otherwise specified

*Males*	**Internal organs**				**Skeletal injuries**			
	*Head neck*	*Thorax*	*Abdomen pelvis*	*MultipleNOS*	*Vert. Column*	*Pelvic girdle*	*Limbs*	*Total*

00 > 00	3.8	0.0	0.3	0.7	0.0	0.0	0.0	4.9
01 > 04	1.9	0.4	0.1	0.5	0.0	0.0	0.0	2.9
05 > 14	2.3	0.3	0.1	1.0	0.0	0.1	0.0	3.8
15 > 24	14.3	3.3	0.9	9.9	0.2	0.0	0.1	28.7
25 > 34	18.4	4.6	1.0	13.5	0.4	0.1	0.1	38.1
35 > 44	14.1	4.1	0.9	10.7	0.3	0.1	0.1	30.2
45 > 54	13.3	4.0	0.9	9.3	0.2	0.2	0.3	28.2
55 > 64	14.6	4.4	1.0	9.2	0.2	0.3	1.0	30.7
65 > 74	29.9	9.2	1.5	14.7	1.5	0.9	9.5	67.1
75 > 84	55.6	10.1	2.3	20.8	1.7	4.7	63.8	159.1
85 > 120	144.1	17.7	7.0	41.8	4.2	28.4	651.1	894.2
total	16.8	4.2	0.9	10.0	0.4	0.6	8.8	**41.6**
								
*Females*	**Internal organs**				**Skeletal injuries**			

	*Head neck*	*Thorax*	*Abdomen pelvis*	*MultipleNOS*	*Vert. Column*	*Pelvic girdle*	*Limbs*	*Total*

00 > 00	1.1	0.0	0.0	0.0	0.0	0.0	0.0	1.1
01 > 04	0.7	0.1	0.0	0.3	0.0	0.0	0.0	1.0
05 > 14	0.9	0.3	0.2	0.6	0.0	0.0	0.0	2.0
15 > 24	3.4	0.5	0.2	2.5	0.0	0.1	0.1	6.8
25 > 34	3.4	0.7	0.1	2.9	0.1	0.0	0.0	7.2
35 > 44	2.9	0.6	0.3	2.7	0.1	0.0	0.0	6.6
45 > 54	3.6	0.7	0.2	2.7	0.1	0.1	0.2	7.6
55 > 64	3.4	1.1	0.2	3.2	0.1	0.1	1.0	9.2
65 > 74	8.5	1.7	0.4	4.9	0.1	0.5	8.3	24.4
75 > 84	21.2	2.8	1.0	7.4	0.4	3.8	73.5	110.1
85 > 120	74.2	6.9	2.2	12.1	1.0	38.2	697.7	832.2
total	5.8	1.0	0.3	3.1	0.1	1.0	17.9	**29.2**
								
*Total*	**Internal organs**				**Skeletal injuries**			

age	*Head neck*	*Thorax*	*Abdomen pelvis*	*MultipleNOS*	*Vert. Column*	*Pelvic girdle*	*Limbs*	*Total*

00 > 00	2.5	0.0	0.2	0.4	0.0	0.0	0.0	3.1
01 > 04	1.3	0.2	0.0	0.4	0.0	0.0	0.0	2.0
05 > 14	1.6	0.3	0.1	0.8	0.0	0.0	0.0	2.9
15 > 24	9.0	1.9	0.5	6.3	0.1	0.0	0.1	17.9
25 > 34	10.9	2.7	0.6	8.2	0.2	0.1	0.1	22.7
35 > 44	8.5	2.3	0.6	6.7	0.2	0.1	0.1	18.4
45 > 54	8.4	2.3	0.6	5.9	0.1	0.2	0.3	17.7
55 > 64	8.8	2.7	0.6	6.1	0.1	0.2	1.0	19.5
65 > 74	17.9	5.0	0.8	9.2	0.7	0.7	8.9	43.1
75 > 84	34.4	5.7	1.5	12.5	0.9	4.2	69.8	128.9
85 > 120	94.9	10.1	3.6	20.9	1.9	35.3	683.9	850.6
total	11.2	2.5	0.6	6.4	0.2	0.8	13.5	**35.3**

**Table 4:** Trauma deaths from hospital discharge registry in Italy. Patients have been selected using ICD9-CM codes 800.0 – 939.9 and 950.0 – 959.9 and AIS ≥ 3 or more.

Age	Males	Females	total
0–14	46	0	46
15–29	583	45	627
30–49	467	277	744
50–69	1,008	421	1,429
≥70	3,241	3,783	7,024
total	5,345	4,526	**9,871**

Bias associated with the use of existing databases are those derived from using data which have been recorded for alternate purposes. In hospitals discharge databases only five or six injuries and procedures are coded and sequenced using ICD-9-CM, to provide maximal payment in the Disease-Related Groups model[27]. Stratification of severity of injury may be accomplished by conversion of ICD-9-CM codes into Abbreviated Injury Scale codes, using appropriate conversion programs[28].

Because of these limitations all these studies are classified as providing "high end" Class III evidence[24]. However, owing to the large sample size and the almost complete sample capture, population-based studies provide the strongest evidence regarding the effects of trauma systems and trauma centers on patient outcomes. Magnitude of benefit after trauma system implementation is an approximately 15% to 20% reduction in preventable trauma deaths, especially among patients with more serious injuries[29-34]. Recently [35], using hospital discharge registries of 69 hospitals of United States, it has been confirmed that risk of death for severe trauma is significantly lower when care is provided in a trauma center than in a non-trauma center.

## Conclusion

Trauma care is a serious, ubiquitous, and expensive health care problem for our society. Nowadays, funding for trauma is still low, especially in some European countries, when compared with other fields of medical research. A death following a traumatic event, as a car accident or a fall at work, is often perceived by population as an unavoidable fatality. Preventable death studies, using panel reviews, registry comparisons, or population-based registries, have uniformly and clearly demonstrated a significant decline in PDR after implementation of trauma systems organized on a regional base. Because of bias of various methodologies none of these researches has provided class I or class II evidence in support of trauma systems. But we should not apologize: preventable death studies are the best available methodology that we have in this field: they have been useful to create the collective conscience how to learn from our mistakes and have given impulse to the development of many organized trauma systems.
